# Invasive fungal infections caused by rare yeast-like fungi in adult patients: results of a prospective study

**DOI:** 10.22034/cmm.2025.345265.1559

**Published:** 2024-12-31

**Authors:** Sofya Khostelidi, Olga Kozlova, Elena Shagdileeva, Ekaterina Burygina, Yulia Borzova, Tatyana Bogomolova, Anastasia Taraskina, Natalya Vasilyeva

**Affiliations:** 1 Department of Clinical Mycology, Allergology and Immunology, North-Western State Medical University named after I.I. Mechnikov, St. Petersburg, Russia; 2 Kashkin Research Institute of Medical Mycology, North-Western State Medical University named after I.I. Mechnikov, St. Petersburg, Russia

**Keywords:** *Trichosporon* spр., *Rodotorula* spр., rare invasive mycoses, yeast-like fungi

## Abstract

**Background and Purpose:**

Fungal infections caused by rare pathogens are becoming an increasingly pressing problem in modern healthcare due to the severe course of the disease, high incidence of disability and mortality of patients. To study clinical and laboratory features and treatment of severe fungal infections caused by rare yeast-like pathogens in adult patients.

**Materials and Methods:**

The prospective observational non-interventional study (2004-2022) included 310 adult patients with severe fungal infections in the Kashkin Research Institute of Medical Mycology based on North-Western State Medical University named after I.I. Mechnikov, Saint-Petrsburg, Russian Federation (from October 2004 to December 2022). To identify the pathogen, we used direct microscopy, microscopy with calcofluor white, culture isolation from blood and tissue biopsies, cerebrospinal fluid or BAL fluid. Micromycete cultures were identified to species based on morphological characteristics and PCR-test.

**Results:**

We treated 310 adult patients with severe fungal infections -10% of them caused by rare yeast-like pathogens (n=30). Analysis of the data presented a general portrait of the patient: a 30-year-old man who has been in the ICU for more than 14 days (93%). Most often, the pathogen was isolated from the blood or biofilm of the central venous catheter (77%). Isolated damage to organs and tissues (without fungemia) was diagnosed in 23% of patients (involving the central nervous system, lungs and skin). *Trichosporon* spр. and Rhodotorula spр. were the main pathogens (together - 73%). Despite treatment, mortality remains very high - 37%.

**Conclusion:**

It is necessary to examine the biological substrate from the lesion daily for fungi if there is no effect from standard therapy. It is necessary to perform species identification of the pathogen and determine sensitivity to antimycotics.

## Introduction

Invasive fungal infections are a growing problem in critically ill patients and are associated with increased morbidity and mortality [ 1 ]. Most of them are due to Candida and Aspergillus species. Successful treatment of these infections and the use of antifungal prophylaxis have led to the emergence of breakthrough fungal infections caused by rare yeast-like fungi (*Trichosporon* spр., *Rhodotorula* spр., *Geotrichum* spр., *Saсcharomyces* spр., *Malassezia* sp.).

“Rare” invasive fungal infection (IFI) is 1 case in 100,000 people per year [ 2 ]. This group IFI included non-Aspergillus spp., non-Candida spp., non-Cryptococcus spp. and non-*Pneumocystis jirovecii*, as well as non-endemic invasive mycosis. We have registered all our patients with rare invasive mycoses in the special database «Data Register Department of Clinical Mycology, Allergology and Immunology of the North-Western State Medical University named after I.I. Mechnikov» [ 3 ]. We analyzed data from patients with rare invasive mycoses caused by yeast-like pathogens.

The study aims to investigate the etiology, clinical manifestations, risk factors, underlying diseases, diagnostic methods, and treatment efficacy of severe fungal infections caused by rare yeast-like pathogens in adult patients. 

## Materials and methods

The study was conducted in accordance with the recommendations of the Helsinki Declaration and approved by the Local Ethics Committee of the North-Western State Medical University. I.I. Mechnikov, Saint Petersburg, Russia. All participants signed informed consent.

The prospective, observational, non-interventional study included 30 patients with rare yeast-like invasive fungal infections from in the Kashkin Research Institute of Medical Mycology based on North-Western State Medical University named after I.I. Mechnikov, Saint-Petrsburg, Russian Federation (from October 2004 to December 2022). The study was conducted in accordance with the recommendations of the Declaration of Helsinki and approved by the local ethics committee of the North-Western State Medical University. I.I. Mechnikov, St. Petersburg, Russia (Protocol dated 20.11.2020). All participants signed an informed consent form.

Clinical and laboratory criteria EORTС/MSGERC, 2020 were used for the diagnosis of IFI [ 5 ]. «Probable» invasive mycosis required the presence of at least: 1 host factor, clinical signs and mycological data (microscopy and/or culture of normally sterile substrates). «Proven» invasive mycosis required a positive histological examination.

Host factors included: ICU, CVC, neutropenia (<500 neutrophils/mm^3^ for >10 days); hematologic malignancy; receipt of allogeneic stem cell transplantation; prolonged use of corticosteroids (≥0.3 mg/kg prednisolone for ≥3 weeks in the past 60 days); treatment with other T-cell immunosuppressants in the past 90 days; treatment with B-cell immunosuppressants; acute graft-versus-host disease grade III or IV involving.

Inclusion criteria: «probable» or «proven» rare yeast-like invasive fungal infections.

*Exclusion criteria*: «probable» or «proven» invasive candidiasis or other fungal infection, including pneumocystis pneumonia, invasive aspergillosis or mucormycosis. 

To identify the pathogen, we used direct microscopy, microscopy with calcofluor white, culture isolation from blood and tissue biopsies, cerebrospinal fluid or BAL fluid. Micromycete cultures were identified to species based on morphological characteristics.

Yeast isolates were identified to the genus level by morphology and to the species level by MALDI-TOF mass-spectrometry (Bruker Daltonics). Species identification of the isolated strains of rare yeast-like fungi was confirmed by sequencing of the internal transcribed spacer (ITS) rDNA region using fungal general primers: ITS1 (5`-TCCGTAGGTGAACCTGCGG-3`) and ITS4 (5`-TCCTCCGCTTATTGATATGC-3`) [ 4 ]. 

The Sequential Organ Failure Assessment (SOFA) scores were calculated to assess the severity of the disease and to predict the clinical outcomes. The tool was based on six criteria (score ranges from 0 to 4 for each) reflecting the function of the organ systems (respiratory, cardiovascular, renal, neurological, hepatic and coagulation). The total score ranged from 0–24. The higher was the score the greater was the insufficiency of the assessed systems and the degree of multiorgan dysfunction.The obtained biomedical data were processed using the software system STATISTICA for Windows (version 13.0). Quantitative data were processed using statistical means. An odds ratio (OR) calculation system was used to assess the possible influence of background diseases and risk factors on the development of infection. An OR value >1 was considered significant. Taking into account the characteristics of the data collection, non-parametric methods were used for statistical analysis: Mann-Whitney test, and discrete variables (Chi-square and Fisher's exact test). The p-value was considered as a criterion of statistical reliability of the obtained results. When p>0.05, the influence of the sign was considered insignificant. Survival was analyzed by the Kaplan-Meier method. The influence of various factors (demographics, background conditions, clinical options and treatment tactics) on survival was evaluated.

The PubMed, Web of Science databases and www.aspergillus.org.uk were analysed using the following words: *Trichosporon* spр., *Rhodotorula* spр., *Geotrichum* spр., *Saсcharomyces* spр., *Malassezia* sp., hematological, ICU, invasive rare mycoses, yeast-like fungi.

## Results

The study included 30 adult patients with «proven» and «probable» rare yeast-like invasive fungal infections (EORTC/MSGERC, 2020). The age of patients ranged from 20 to 61 years, the median was 30 years [23;41] and males were 60% (Table 1). 

**Table 1:** Data of adult patients with Invasive fungal infections caused by rare yeast-like pathogen and control

Characteristics of patients		IFI (rare yeast-like pathogen) (N=30)		Severe patients without rare yeast-like pathogen IFI (N=30)		P-value	OR
		n	%	N	%		
Baseline	Males	18	60	18	60	0,991	
	Females	12	40	12	40	0,987	
	Аge of patients, median	30 [23;41]		31 [26;40]		0,961	
Underlying diseases	Hematological diseases	15	50%	15	50%	0,537	
	Acute leukemias	8	27% (54% from 15)	8	27% (54% from 15)	0,487	
	Polytrauma/burns	5	17%	6	20%	0,487	
	AIDS	4	13	2	7%	0,101	
	Solid tumors	3	10%	3	10%	0,105	
	Pancreatic necrosis	2	7%	3	10%	0,231	
	Cirrhosis of the liver	1	3%	1	3%	0,182	
Risk factors	Bacterial sepsis	26	87	22	73	0,204	<1
	CVC	29	97	27	90	0,312	<1
	CVC, Me days	16 [14;18]		10 [10;12]		0,00001*	
	CVC more than 14 days	26	90	4	15	0,00001*	42,3
	ICU	29	97	22	72	0,011*	7
	ICU, Me days	12 [10;20]		11 [7;15]		0,481*	
	SOFA, Me	9 [7;10]		6 [5;8]		0,017*
	SOFA 7	15 (n=20)	75	6 (n=16)	37	0,027*	5
	ICU 14 days	26 (n=28)	93	4 (n=20)	20	0,00001*	52
	Mechanical ventilation	14	47	7	35	0,062	<1
	GCS	22	73	15	50	0,008*	<1
	GCS, Me mg	84 [56;140]		28 [28;80]		0,00001*	
	GCS, Me days	4 [1;10]		2 [1;4]		0,008*
	GCS, 60 mg	14 (n=22)	63	6 (n=15)	40	0,001*	<1
	GCS, 10 days	6 (n=22)	27	1 (n=15)	7	0,014*	<1
	PCT	15	50	13	43	0,613	<1
	Number of PCT courses	5 [2;8]		2 [1;5]		0,001*	
	Allo-HSCT	3	10	1	3	0,312	<1
	GVHD	3	10	1	3	0,312	<1
	Lymphocytopenia < 1,0×10^9^/l	20	67	7	23	0,001*	6,6
	Lymphocytopenia, Me days	19 [15;26]		7 [2;17]		0,100	
	Lymphocytopenia 14 days	15 (n=20)	75	3 (n=7)	43	0,00001*	<1
	Agranulocytosis	14	47	8	27	0,067	<1
	Agranulocytosis, Me days	16 [14;20]		4 [2;7]		0,00001*	
	Agranulocytosis 14 days	8 (n=15)	53	1 (n=8)	13	0,038	<1
	Antimycotic prophylaxis	8	27	21	70	0,001*	<1

* р <0,05, n - is the number of patients with an identified risk factor; N - is the total number of patients in the study group with data available, AIDS - Acquired Immune Deficiency Syndrome; allo-HSCT - allogeneic hematopoietic stem cell transplantation; CVC- central venous catheter; GVHD - graft-versus-host disease; GCS – glucocorticosteroids; ICU- intensive care unit; Me- mediana, PCT – polychemotherapy.

The control group - with the same demographics and similar background conditions in the ICU or specialized wards for severe hematological patients were formed. patients of control group consisted of 30 adult patients from the ICU or specialized wards for severe hematological patients without fungal infections with median age 31 [26; 40] years, males – 60%.

The main underlying conditions in patients with IFI caused by rare yeast-like micromycetes were hematological diseases (50%, n=15), polytrauma/burns (17%, n=5), and AIDS (13%, n=4). Less common underlying conditions were solid tumors, pancreatic necrosis and liver cirrhosis.

The main risk factors were: CVC more than 14 days (90% vs 15%, p< 0.0001, OR=42.3), ICU more than 14 days (93% vs 20%, p< 0.0001, OR=52), the severity of the general condition - SOFA 9 points or more (9 vs 6, p = 0.011). If SOFA was more than 7, the risk of developing IFI caused by rare yeast-like fungi increased (OR=5).

We analyzed risk factors in hematological patients and patients with other underlying conditions (Tables 2 and supplementary Table 1).

**Table 2:** Risk factors invasive fungal infection caused by rare yeast-like pathogens in non-hematological patients

Risk factors	Non-hematological patients with IFI (with rare yeast-like pathogen) (N=15)		Severe non-hematological patients without IFI (N=15)		p-value	ОR
	N	%	n	%		
Surgical	11	73	12	80	0,498	<1
Bacterial sepsis	13	87	10	67	0,631	<1
CVC	15	100	15	100	0,537	<1
ICU	15	100	15	100	0,105	<1
SOFA	9 [7;11]	6,5 [4;8,5]		0,040*	
ICU, days	25 [22;29]		12,5 [10;26]		0,048*	
ICU 14 days	11 (n=13) 85%	7 (n=15) 47%		0,032*	
Mechanical ventilation	12	80	6	40	0,101	6.0
GCS	7	47	1	7	0,022*	NA
GCS, mg	156 [30;300]		20			
GCS, days	4 [0;5]	10			
Lymphocytopenia < 1,0х10^9^/л	6	40	0			
Lymphocytopenia, days	19 [12;25]	-			
Agranulocytosis	1	7	0			
Antimycotic prophylaxis	2	13	14	93	0,000*	9.1
Empirical therapy	0	-	7	43		

* р <0.05

Risk factors in non-hematological patients were: bacterial sepsis (87% vs 67%), long stayed in ICU (24.5 days vs 12.5 days, p=0.048), SOFA 9 (9 vs 6.5 points, p=0.039-), used GCS (47% vs 7%, p = 0.022) and antifungal prophylaxis (87% vs 7%, p < 0.001) (tab. 3). Echinocandins for empirical administration used in control group patients (43% vs 0).

Fungemia was the main clinical variant (80% vs 73%). The main localization was: skin and soft tissues (20% vs 0), lungs (20% vs 0), central nervous system (7% vs 33%), heart (0 vs 7%), peritonitis (0 vs 7%). Skin lesions were observed in three patients with fungemia (Table 3).

**Table 3:** Clinical variants and Etiology of invasive fungal infection caused by rare yeast-like pathogens

		Patients with IFI caused by rare yeast-like pathogen (N=30)		Hematological patients with IFI, caused by rare yeast-like pathogen (N=15)		Non-hematological patients with IFI, caused by rare yeast-like pathogen (N=15)		p-value
		N	%	N	%	n	%	
Clinical variants	Fungemia23	77	12	80	11	73	0,498
	Skin and soft tissues	3	10	3	20	0	-	
	Lungs	3	10	3	20	0	-	
	Brain	6	20	1	7	5	33	0,094
	Sinuses	1	3	1	7	0	-	
	Heart (endocarditis)	1	3	0	-	1	7	
	Peritonitis	1	3	0	-	1	7	
	Dissemination (2 or more organs)	9	30	6	40	3	20	0,107
Etiology	*Trichosporon*/ *Cutaneotrichosporon* spр.	12	40	5	33	7	47	
	*Rhodotorula* spр.	10	33	5	33	5	33	
	*Magnusiomyces*/ *Geotrichum* spр.	5	17	3	20	2	13	
	*Saccharomyces* spp.	2	7	1	7	1	7	
	*Malassezia* spp.	1	3	1	7	0	-	

Damage to 2 or more organs was more common in hematologic patients (40% vs 20%).

Clinical manifestations of infections were nonspecific and depended on location. The main symptom was body temperature above 38.5oC (100% vs 93%, p=0.332). Some patients also complained of cough, hemoptysis, and local pain (Table 3).

The diagnosis was made on the basis of inoculation of the pathogen in normally sterile biosubstrates (blood, cerebrospinal fluid, abdominal fluid, skin and mucous tissue biopsies).

The main causative agents of severe fungal infections caused by rare yeast-like micromycetes were: *Trichosporon/Cutaneotrichosporon* spp. (40%), *Rhodotorula* spp. (33%), *Magnusiomyces/Geotrichum* spp. (17%), *Saccharomyces* spp. (7%) and *Malassezia* spp. (3%). No significant differences in the etiology of severe rare yeast-like mycotic infections between hematological and “other” patients were identified (Table 3).

Eleven yeast-like pathogens were identified to the species level: *Trichosporon asahii* (4 cases), *Magnusiomyces capitatus* (3 cases), *Cutaneotrichosporon mucoides* (1 case), *Saccharomyces cerevisiae* (1 case), *Rhodotorula mucilaginosa* (1 case), *Malassezia furfur* (1 case).

Аntimycotic therapy received 87% of patients (in four cases the diagnosis was made posthumously).

The most frequently used drug in both groups was amphotericin B (AmB) deoxycholate (57% vs 58%, p=0,615). Less commonly prescribed were echinocandins (29% vs 8%, p=0,137), voriconazole (29% vs 17%, p=0,276), itraconazole (14% vs 0), posaconazole (7% vs 0), lipid forms of amphotericin B (7% vs 0). The progression of IFI required the prescription of combination antimycotic therapy (21% vs. 17%, p=0,599). The following combinations of antimycotics were used: voriconazole and echinocandins, voriconazole and amphotericin B, amphotericin B and fluconazole (Table 4). The mortality of patients was assessed after 4 weeks and 12 weeks from the moment of diagnosis of the myсotic infection and the start of antifungal therapy. The mortality of patients with severe rare fungal infections caused by yeast-like micromycetes over 4 weeks was 37% (Supplementary Figure 1). 

**Table 4:** Treatment of invasive fungal infection caused by rare yeast-like pathogens

	Patients with IFI caused by rare yeast-like pathogen (N=30)		Hematological patients with IFI, caused by rare yeast-like pathogen (N=14)		Non-hematological patients with IFI, caused by rare yeast-like pathogen (N=12)		p-value
	n	%	n	%	n	%	
AmB deoxycholate	15	50	8	57	7	58	0,615
Echinocandins	5	33	4	29	1	8	0,137
Voriconazole	6	20	4	29	2	17	0,276
Fluconazole	16	53	4	29	12	100	0,009*
Itraconazole	2	7	2	14	0	-	
Posaconazole	1	3	1	7	0	-	
AmB lipid complex	1	3	1	7	0	-	
Combination therapy	5	33	3	21	2	17	0,599
Antimycotic therapy, days (Me)	33 [8;81]		33 [8;81]		17 [4;57]		0,322
Surgery	17	57	7	50	10	83	0,107

* р <0,05, AmB; Amphotericin B, IFI; invasive fungal infection

The average life expectancy of patients was 4 vs 2 weeks. There were no significant differences in overall survival in the groups of hematological patients and those with “other” background conditions (Table 5).

**Table 5:** Invasive fungal infection caused by rare yeast-like pathogens – mortality

Survival	Patients with IFI caused by rare yeast-like pathogen (n=30)		Hematological patients with IFI, caused by rare yeast-like pathogen (n=15)		Non-hematological patients with IFI, caused by rare yeast-like pathogen (n=15)		p-value
	n	%	n	%	n	%	
4 weeks	19	63	11	73	9	60	0,113
12 weeks	11	37	5	33	6	40	0,498
Survival, weeks (Me)	3 [0;12]		4 [2;12]		2 [0;12]		0,948

IFI; invasive fungal infection

**Figure 1:**
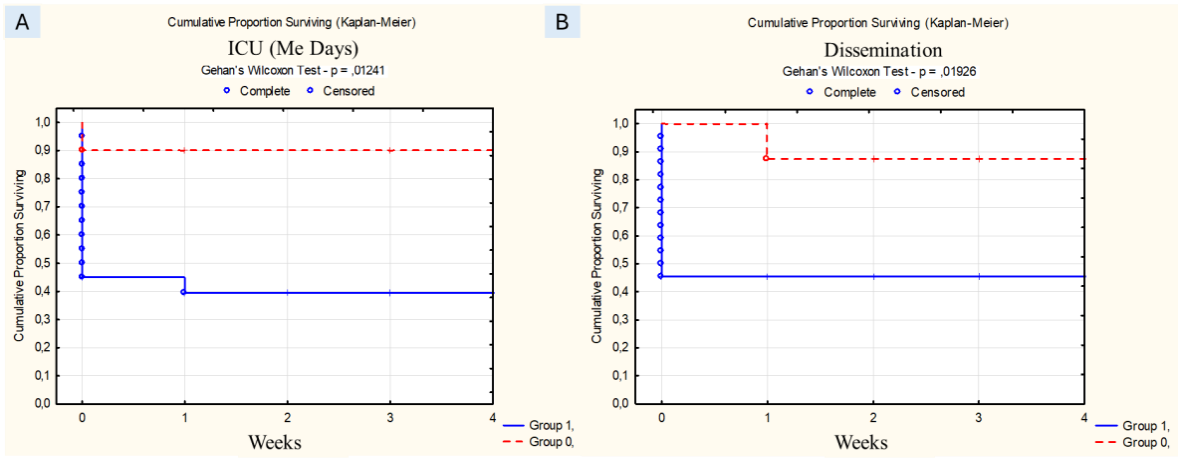
A) ICU for more than 14 days (group 1 - with sign, group 0 - without sign); В) dissemination (group 1 - with dissemination, group 0 - without dissemination).

Analysis of the influence of background diseases, risk factors and other factors on the survival of patients showed that the prognosis was unfavorable for the severity of the condition of patients in the ICU (SOFA “9” points or more, 86% vs 46%, p = 0.01114) and long stay in the ICU (more than 7 days, 90% vs 40%, p=0.01241), fungemia (87% vs 45%, p=0.01926) (Figure 1).

Early antimycotic therapy (64% vs 20%, p = 0.04), the use of AmB and AmB lipid forms as initial therapy (73% vs 40%, p = 0.0457) and changed CVC (77.7% vs 18%, p= 0.00369) were well for prognosis (Supplementary Figure 2).

## Discussion

Invasive mycoses associated with rare yeast-like pathogens are most often associated with *Malassezia*, *Pseudozyma (Moesziomyces)*, *Rhodotorula*, *Sporobolomyces*, *Trichosporon*, *Geotrichum*, *Kodamaea*, *Saccharomyces* and *Saprochaete*. These microorganisms can be found in food and water and may be normal human microbiota. But in immunosuppressed patients they cause severe infections associated with a high risk of death.

Moreover, all non-*Сandida* mycoses are about 1-2% of invasive mycotic infections. Thus, in Denmark, rare invasive mycoses together accounted for only 1.1% of 4000 cases of fungal infection over 8 years [ 6 ], while in China up to 1.7% [ 7 ]. In Spain, during a 2-year follow-up, the incidence of invasive mycoses caused by rare pathogens was 1.8% [ 8 ]. Another large study analyzed 2155 isolates of yeast-like pathogens obtained from blood over the course of a year and showed that the cause of rare mycoses was mainly: *Trichosporon* spp. (1.1%), *Rhodotorula* spp. (0.5%), *Kodamaea ohmeri* (0.3%) and *Malassezia* spp. (0.2%) [ 9 ].

In Russia, isolated cases of the development of mycoses caused by rare yeast-like fungi have been described. A general analysis of background diseases and conditions, risk factors, clinical manifestations, diagnosis and effectiveness of treatment has not been done previously [ 3 , 10 , 11 ]. Our study showed that in Russia IFIs associated with rare yeast-like infections occur in departments and intensive care units where patients stayed for more than 14 days (р=0,000*) Often IFIs occur in hematological patients and are associated with a venous catheter. 

According to international data, the main risk factor for the development of rare invasive mycoses associated with rare yeast-like fungi was long-term use of CVCs. Li H. and co-investigators analyzed 140 patients with trichosporonosis in 2020. The use of a CVC was noted in 43% and 20% of patients who were in the ICU [ 12 ]. Kontoyiannis et al. analyzed trichosporosis patients with a history of cancer and found that CVCs were used in 71% of patients [ 13 ].

We have shown that the risk of infections associated with rare yeast-like pathogens increases also on the background of lymphocytopenia (p=0.001) and tends to increase with agranulocytosis (p=0.067). The influence of agranulocytosis and lymphocytopenia on the development of infection is more relevant in the group of hematologic patients. This became evident when comparing the two groups depending on the background disease. In the group of hematologic patients, agranulocytosis lasting more than 14 days (p=0.008*) and lymphocytopenia (p=0.035*) were reliable risk factors. We did not obtain a reliable difference for lymphocytopenia of more than 14 days because of the small number of observations.

International observations also indicate that mycotic infections associated with rare yeast-like fungi are more often associated with hematologic diseases. Thus, 87% of cases of invasive mycoses caused by fungi of the genus *Rhodotorula* were associated with hematoblastosis and solid neoplasms [ 14 , 15 ]. Cases of rhodotorulosis on the background of rheumatologic pathology have also been described [ 16 ]. Published cases of geotrichosis and malasseziosis also developed mainly against the background of hematoblastosis [ 17 - 19 ]. In Russia, isolated cases of infections caused by rare mycosis pathogens have been described, mainly among oncohematologic and HIV-infected patients [ 20 - 22 ].

Most rare yeast-like fungi cause disease in immunocompromised patients and are associated with intravenous catheters or other invasive devices. Fungemia with or without skin lesions develop most commonly. Similar clinical variants develop in rhodotorulosis. *Rhodotorula* spp. was isolated in 4.2% of 8821 clinical specimens in a global study [ 23 ]. The infection was predominantly fungemia (up to 79%), less frequently endophthalmitis (7%), peritonitis (5%) (as a complication of peritoneal dialysis) [ 14 ]. Cases of endocarditis and meningitis were described [ 24 - 26 ]. Trichosporonosis in hematologic patients most often develops in the type of fungemia, often with skin lesions (18-43%) or pneumonia (18-53%) [ 27 - 30 ]. Endocarditis, spleen and liver abscesses are also possible [ 30 , 31 ]. Malasseziosis, like other yeast-like mycoses, is more often fulminant with secondary dissemination to other organs (heart, spleen, brain, lungs) [ 32 , 33 ], cases of osteomyelitis and deep skin lesions with adjacent tissues have been described [ 34 , 35 ].

In our study we observed mostly fungemia (77%), but also brain (20%), lung (10%), sinus (3%), soft tissue and skin lesions (10%), endocarditis (3%) and peritonitis (3%).

Diagnosis of invasive mycoses associated with rare yeast-like fungi is difficult because these are very severe patients with the median SOFA score is 9 [ 3 , 11 ]. In addition, laboratory diagnosticians must have good experience to isolate and identify the culture. In our study as in most published observations the main pathogens were *Trichosporon* spp. (40%), *Rhodotorula* spp. (33%), *Magnusiomyces/Geotrichum* spp. (17%), *Saccharomyces* spp. (7%) and *Malassezia* spp. (3%). This spectrum of pathogens is similar to the international data [ 11 ].

Determining the species of mycotic pathogen is important most species of rare yeast-like pathogens are multidrug resistant. Xiao et al. (2018) published that 100% of *R. mucilaginosa* isolates were resistant to fluconazole and voriconazole. Other non-*Candida* yeast species showed reduced sensitivity to fluconazole (53.3%), but most were sensitive to voriconazole (94.3%) [ 11 ]. *Rhodotorula* spp. are resistant *in vitro* to echinocandins and azoles. Susceptibility to amphotericin B varies and this agent has no fungicidal activity against *Trichosporon*. Clinical failures have been reported in the treatment of trichosporonosis with amphotericin B, fluconazole and their combinations [ 36 ]. The newer triazoles are more active than fluconazole against *Trichosporon*.

New clinical guidelines recommend amphotericin B (lipid forms) with or without flucytosine [ 37 ] for the treatment of invasive mycoses associated with rare yeast-like pathogens. Voriconazole is the drug of choice in trichosporonosis.

Mortality of patients with disseminated infection and fungemia caused by rare yeast-like fungi remains high despite the use of new antimycotics. In the publication of Yong A. and co-authors provide data on 60-70% lethality in disseminated trichosporonosis [ 38 ]. Analysis of cases of invasive geotrichosis showed 65% mortality, with patients with disseminated form amounting to 61% [ 39 ]. In rhodoturulosis mortality is estimated to be 12 to 20% [ 40 ]. In our study, the overall mortality in infections associated with rare yeast-like micromycetes was 37%. The survival rate depended on the prevalence of infection and its localization and on early administration of antimycotic drugs together with CVC replacement. Early antimycotic therapy (64% vs 20%, p = 0.04), the use of AmB and AmB lipid forms as initial therapy (73% vs 40%, p = 0.0457) and changed CVC (77.7% vs 18%, p = 0.00369) were good for prognosis.

## Conclusion

Non-*Candida* yeasts, although accounting for less than 1% of all fungal infections, require further study due to increasing resistance to antifungal drugs. Early diagnosis and early targeted therapy will improve patient survival.
